# A hydrogel reveals an elusive cancer stem cell

**DOI:** 10.1038/s41419-021-03696-7

**Published:** 2021-04-20

**Authors:** Sonia Melino, Dror Seliktar

**Affiliations:** 1 Department of Chemical Science and Technologies, University of Rome “Tor Vergata”, Rome, Italy; 2 Faculty of Biomedical Engineering, Technion-Israel Institute of Technology, Haifa, 3200003 Israel

**Keywords:** Reprogramming, Cancer stem cells

Biomaterials made from polymers have helped to revolutionize the medical industry through innovative designs of implants and biosensors. Now, materials in the form of hydrogels are making headway in cancer research, where an ever-expanding tool-chest of sophisticated in vitro cell culture platforms is helping scientists to unravel the mysteries of cancer stem cells (SCs)^1^.

Biomedical hydrogels, which are hydrophilic polymer-based biomaterials, are transforming the way in which materials impact the medical field^2^. These unique materials function particularly well at the interface with complex cellular systems^3^, thereby providing new ex vivo pathways to control cellular fate or guide cellular function. Sophisticated hydrogels that mimic properties of biological tissues have opened up new potential for biomedical engineering applications^4^, namely in the burgeoning field of SC therapeutics^5^ and cancer research^6^. Biological or synthetic hydrogels are now routinely produced with design features that can actively manipulate multiple aspects of SC signaling pathways leading to enhanced morphogenesis, proliferation, and differentiation^7–9^. Many of these systems take advantage of biological or bio-mimetic motifs that are incorporated into the hydrogel backbone, to alter response based on known cellular interactions with native extracellular matrix (ECM) molecules. These interactions are often combined with mechanical and biophysical features that resemble those of the SC niche, to enhance differentiation pathways towards myogenesis, chondrogenesis, neurogenesis, and more^10,11^. Now, writing in *Nature Biomedical Engineering*, Tanaka and colleagues^1^ use completely synthetic double network (DN) hydrogels to rapidly reprogram differentiated cancer cells into cancer SCs (CSCs), to identify biomarkers for targeted cancer therapy.

CSCs are an elusive cell that constitute a very small fraction of the overall cell population of a tumor mass. Despite their minute numbers, these cells are capable of leading to the recurrence of cancer vis-à-vis a circulating subpopulation derived from their progeny. Identifying these cells in patients is currently not possible, because no definitive set of biomarkers exists for them. Although targeting of this subpopulation of cells could potentially eradicate cancer recurrence, the development of such therapies has been hampered by the limited availability of primary CSCs with which to study their genotype. Tanaka and colleagues^1^ use their DN hydrogel comprising poly(2-acrylamido-2-methylpropane sulfonic acid) and poly(*N,N’*-dimethylacrylamide) as a tough yet soft substrate on which differentiated cancer cells are cultivated with the aim of reprogramming these cells to their scarcer CSC progenitors. Relying solely on the ability to control the physical properties of their DN hydrogel, they reconstructed key mechanical features of the niche microenvironment that causes induction of stemness in six fully differentiated cancer cell lines. Critically, they showed that this rapid and robust reprogramming was obtained when using primary tumor cells from a human brain cancer glioblastoma patient. They unequivocally demonstrated the usefulness of this technique in terms of deriving a potentially unlimited supply of primary human CSCs for biomarker discovery research.

The natural processes leading to SC reprogramming are complex and involve a multitude of extrinsic and intrinsic signaling in the niche. Until now, the main paradigm involved in the artificially induced SC reprogramming involved the use of transcription factors, with their stability regulation^12^, and/or soluble biochemical agonists. A variety of techniques have emerged to produce ever larger quantities of pluripotent SCs for therapeutic application in translational medicine, including regenerative medicine and cell therapy. Far fewer strategies exist for producing CSC from primary tumor cells. This is likely owning to the complex differential spatiotemporal activation of tyrosine kinase required for this transformation, which appears to be activated by the DN hydrogel’s interactions with cellular surface proteins including the integrin family of cell adhesion molecules, but not E-cadherin or β-catenin. The DN hydrogels were not functionalized with bioactive motifs, suggesting that nonspecific spheroid formation was primarily responsible for the biophysical induction of the cells. Nevertheless, a unique combination of physical properties obtained only with the DN hydrogel, and not with any of the respective single-network hydrogels or plastic dish controls, resulted in a robust reprogramming of the cell spheroids expressing all three stemness markers, namely sox2, Oct3/4, and Nanog.

**Fig. 1:**
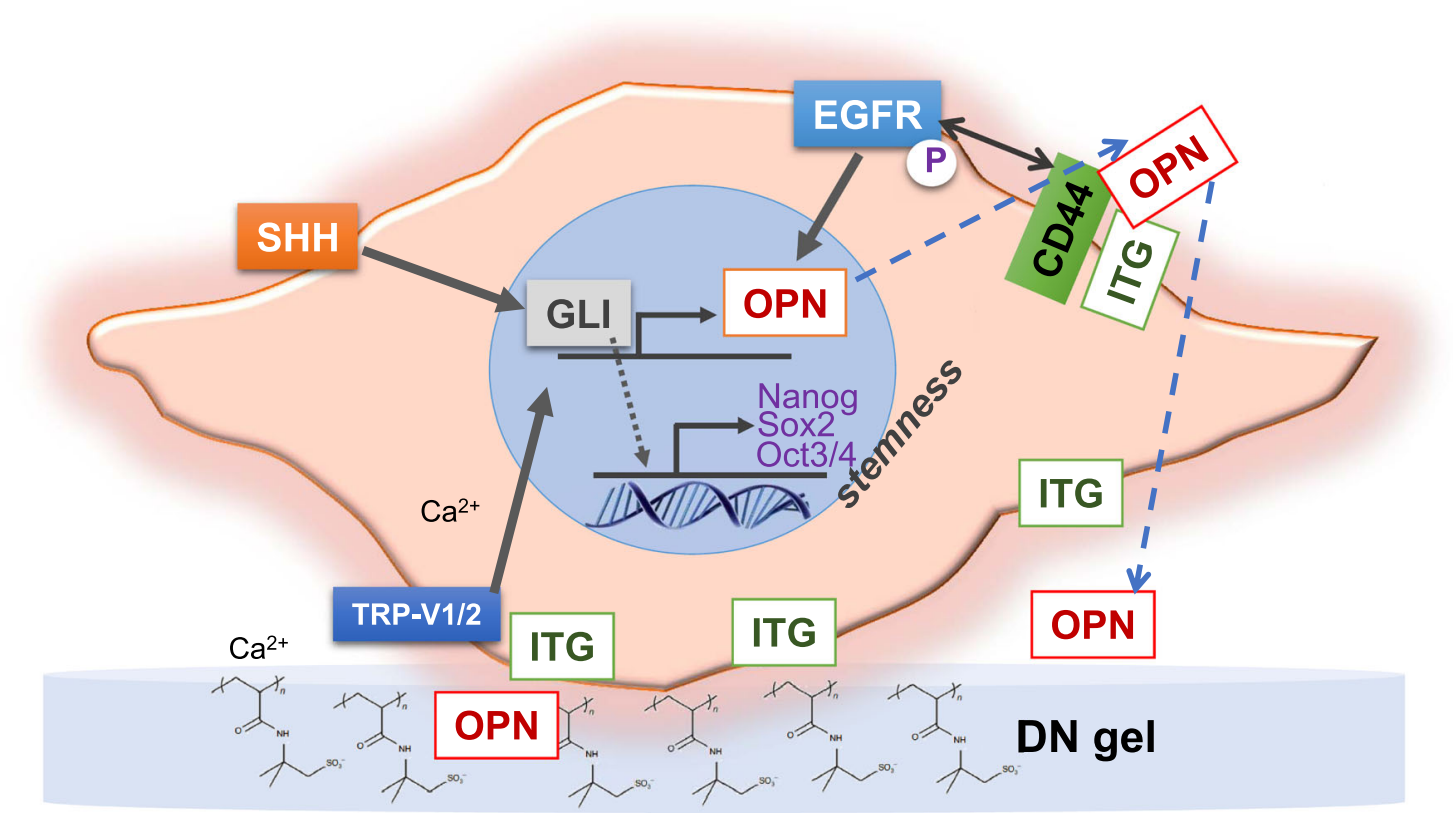
A double network (DN) hydrogel substrate is used to reprogram an elusive cancer stem cell. DN gels seem to be able to induce distinct cellular signals that are able to orchestrate a complex reprogramming of cancer cells via a calcium-dependent transient receptor ion channel potential (TRP), which induces the transcription of osteopontin (OPN) and stemness factors via the transcription factor GLI. Secreted OPN stimulates integrin and CD44 to further induce stemness. See text for further details, as well as the original study by Tanaka and colleagues^1^.

It stands to reason that Tanaka and colleagues^1^ use their DN hydrogel, with its unique set of biophysical properties, as a catalyst to initiate brain cancer cell reprogramming, likely through the production of osteopontin (OPN) and the tyrosine kinase signaling pathways. The OPN causes a signaling feedback loop in these cultures, whereby brain cancer cells are induced to express key proteins related to stemness, such as SHH and GLI1, which in turn act on the OPN-CD44 signaling axis that enhances the stemness in the culture. This positive feedback loop causes a very rapid induction of CSCs on DN hydrogels (Fig. 1), whereas single-network hydrogels used as controls did not catalyze this rapid CSC induction nor the hypothesized reprogramming signaling cascade. Importantly, the DN hydrogel-induced brain CSCs were found to express detectable levels of the platelet-derived growth factor receptor (PDGFR), whereas primary cancer cells express epidermal growth factor receptor but not PDGFR. Targeting the PDGFR receptor on these cells can therefore be used to eradicate this dangerous subpopulation as the authors demonstrated when treating their DN hydrogel brain cell cultures with a PDGFR inhibitor and causing an increase in classic apoptotic marker-cleaved caspase-3.

The utilization of the DN hydrogels in this study demonstrated undeniable synergy when characterizing the molecular and cellular biology of brain CSCs. However, the full utility of these DN hydrogels in SC discovery may be much more profound. The reasons for this are twofold. The ability to precisely control SC reprogramming by matrix-mediated induction has been shown to require a more elaborate understanding of the structure–function relationship by which cells interface with their matrix. The authors pointed out: “that the essential chemical and/or physical factor(s) of the DN gel were not directly described, and further study is required”^1^. Beyond the specific interactions afforded by a two-dimensional hydrogel substrate, such materials can be designed to encapsulate SCs and cellular spheroids in a three-dimensional arrangement, which more closely resembles the niche ECM^4,13,14^. Moreover, when combined with elegant spatiotemporal presentation of biomolecules, the unique combinations of biochemical and biophysical properties may be used to achieve far more versatility from this system^15^. It will be extremely exciting to see other, potentially more far-reaching manifestations of this reprogramming approach evolve systematically, as focus shifts further towards a material design-based strategy.
